# Correction: Validation of stearoyl-coA desaturase gene TaqMan probe-based SNP for genotyping Tattykeel Australian White MARGRA lamb for health-beneficial omega-3 long-chain fatty acids and intramuscular fat content

**DOI:** 10.1371/journal.pone.0356912

**Published:** 2026-08-24

**Authors:** Aduli Enoch Othniel Malau-Aduli, Shedrach Benjamin Pewan, Felista Waithira Mwangi, John Roger Otto

There is an error in the Acknowledgment section. The correct Acknowledgment section is as follows: The authors gratefully acknowledge The Gilmores of Tattykeel Australian White Pty Ltd, Oberon, New South Wales, Australia for access to herd and farm resources, and the Commonwealth Scientific and Industrial Research Organization SIEF Ross Metcalf STEM Business Industrial Research Fellowship programme. We would also like to appreciate the support of the School of Environmental and Life Sciences, College of Engineering, Science and Environment, The University of Newcastle, Callaghan, New South Wales, Australia and James Cook University, Townsville, Queensland, Australia.

## References

[1] Malau-Aduli AEO, Pewan SB, Mwangi FW, Otto JR. Validation of stearoyl-coA desaturase gene TaqMan probe-based SNP for genotyping Tattykeel Australian White MARGRA lamb for health-beneficial omega-3 long-chain fatty acids and intramuscular fat content. PLoS One. 2026;21(1):e0339573. doi: 10.1371/journal.pone.0339573 41493932 PMC12773820

