# Supplementary material for: Validation of stearoyl-coA desaturase gene TaqMan probe-based SNP for genotyping Tattykeel Australian White MARGRA lamb for health-beneficial omega-3 long-chain fatty acids and intramuscular fat content
Source: PLoS One. 2026 Jan 6;21(1):e0339573. doi: 10.1371/journal.pone.0339573 (PMC12773820; doi:10.1371/journal.pone.0339573)
Supplement: S2 Appendix — (DOCX) [file pone.0339573.s002.docx]

**TAW MARGRA lamb cut A**

| 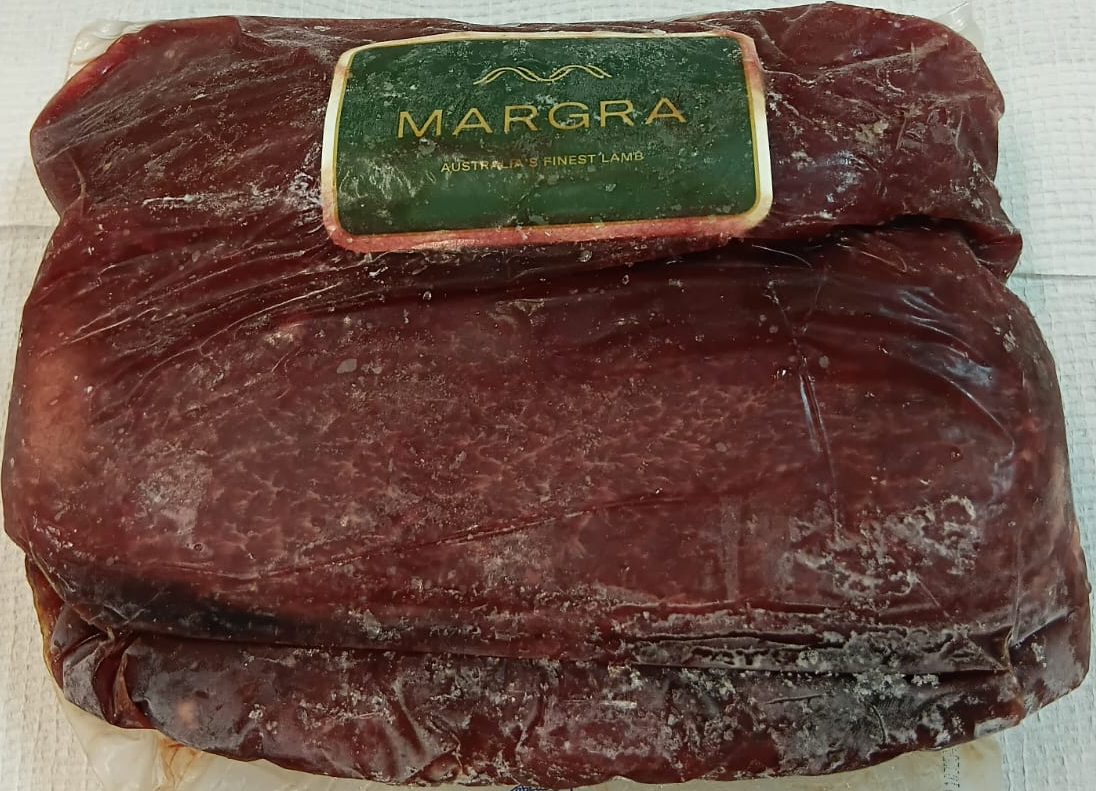 |
| --- |
|  |

**Fat melting point (°C) 35.2 Minerals:**

**Intramuscular fat content (%) 3.9 Iron (mg/100g) 2.5**

**Protein percentage (g/100g) 23.0 Zinc (mg/100g) 2.8**

**Total Fat percentage (g/100g) 6.5 Calcium (mg/100g) 35.2**

**Energy (kJ/100g) 750.2 Phosphorus (mg/100g) 251.2**

**Oleic acid - C18:1ꙍ9 (mg/100g) 3396.0 Magnesium (mg/100g) 30.1**

**ALA α-linolenic acid C18:3ꙍ3 (mg/100g) 179.2 Sodium (mg/100g) 45.7**

**Long-chain omega-3s: Potassium (mg/100g) 322.4**

**EPA - C20:5ꙍ3 (mg/100g) 24.8 Copper (mg/100g) 0.1**

**DHA - C22:6ꙍ3 (mg/100g) 8.0 Selenium (mg/100g) 17.6**

**DPA - C25:5ꙍ3 (mg/100g) 25.4**

**EPA + DHA (mg/100g) 32.8**

**EPA + DHA + DPA (mg/100g) 58.2**

**TAW MARGRA lamb cut B**

| **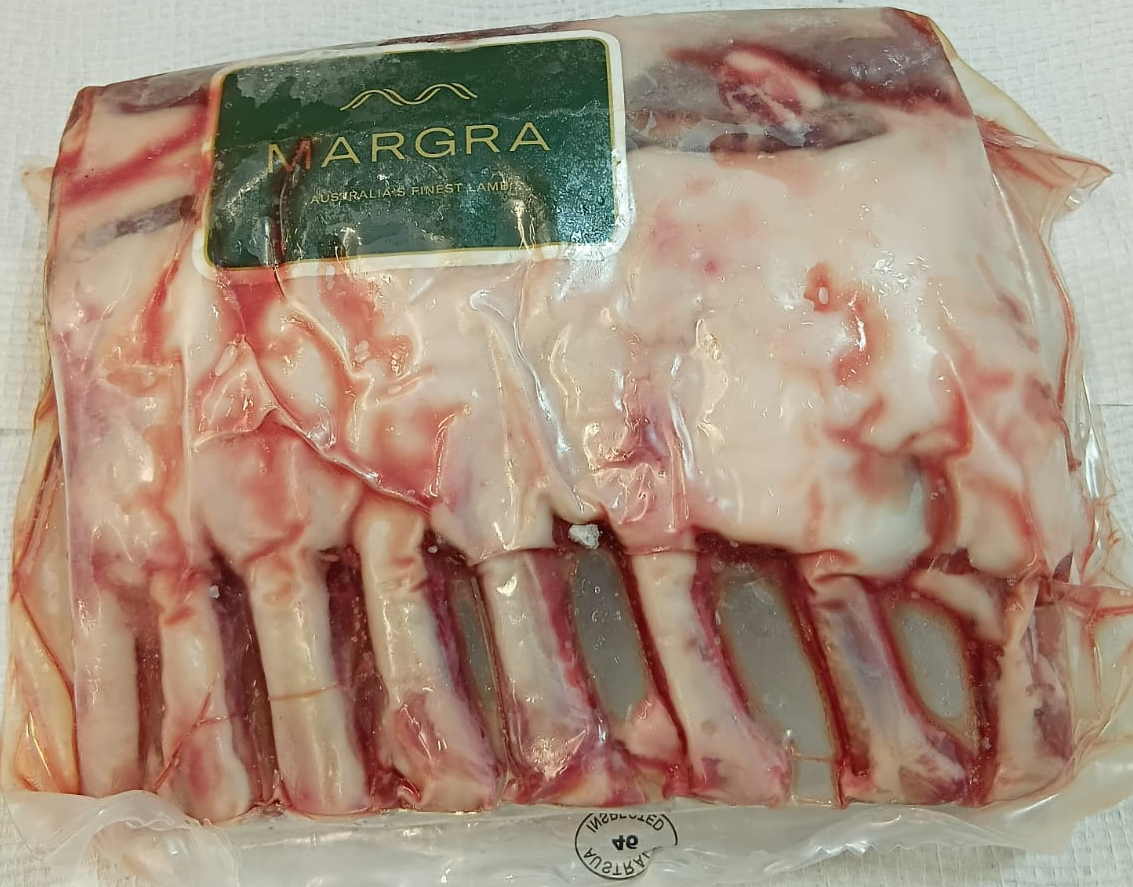** |
| --- |
|  |

**Fat melting point (°C) 36.9 Minerals:**

**Intramuscular fat content (%) 3.6 Iron (mg/100g) 2.9**

**Protein percentage (g/100g) 22.5 Zinc (mg/100g) 2.7**

**Total Fat percentage (g/100g) 13.5 Calcium (mg/100g) 38.2**

**Energy (kJ/100g) 766.2 Phosphorus (mg/100g) 260.2**

**Oleic acid - C18:1ꙍ9 (mg/100g) 2071.5 Magnesium (mg/100g) 30.8**

**ALA α-linolenic acid C18:3ꙍ3 (mg/100g) 98.8 Sodium (mg/100g) 46.7**

**Long-chain omega-3s: Potassium (mg/100g) 325.4**

**EPA - C20:5ꙍ3 (mg/100g) 23.7 Copper (mg/100g) 0.1**

**DHA - C22:6ꙍ3 (mg/100g) 9.0 Selenium (mg/100g) 17.0**

**DPA - C25:5ꙍ3 (mg/100g) 24.4**

**EPA + DHA (mg/100g) 32.7**

**EPA + DHA + DPA (mg/100g) 57.1**

**TAW MARGRA lamb cut C**

| **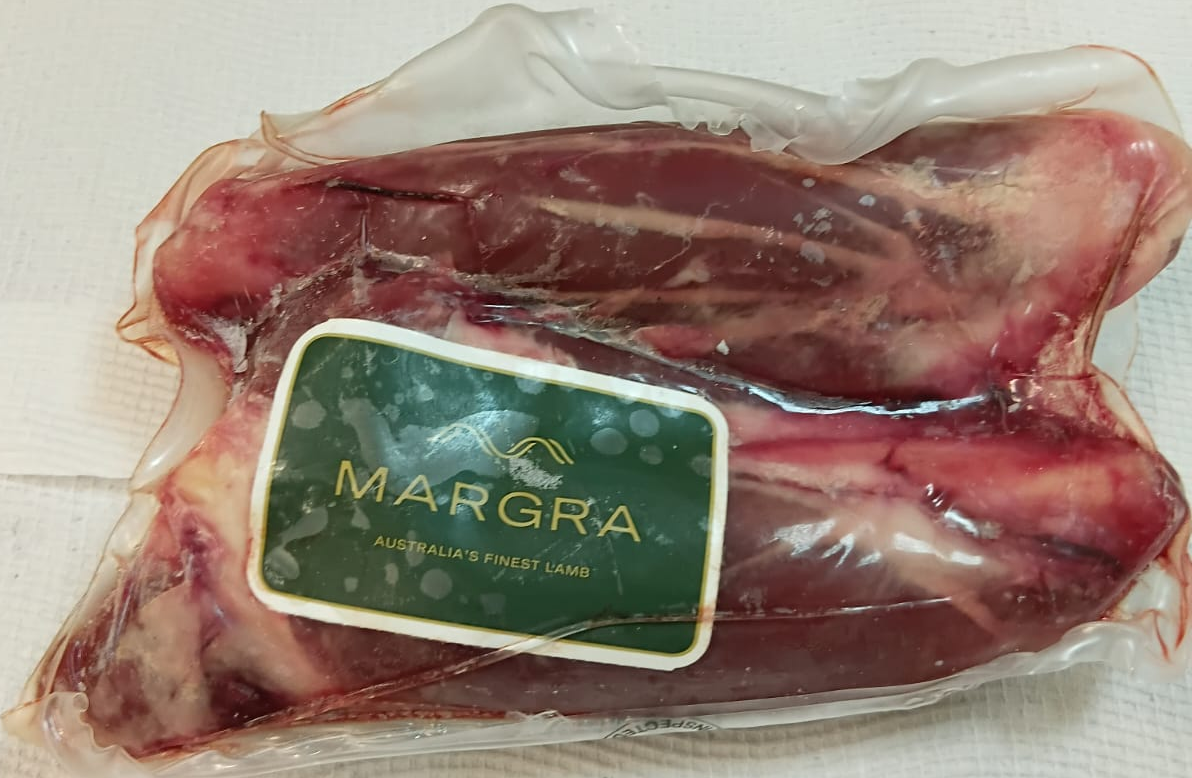** |
| --- |
|  |

**Fat melting point (°C) 35.0 Minerals:**

**Intramuscular fat content (%) 2.9 Iron (mg/100g) 2.6**

**Protein percentage (g/100g) 23.2 Zinc (mg/100g) 2.5**

**Total Fat percentage (g/100g) 6.0 Calcium (mg/100g) 36.7**

**Energy (kJ/100g) 738.2 Phosphorus (mg/100g) 255.3**

**Oleic acid - C18:1ꙍ9 (mg/100g) 3204.6 Magnesium (mg/100g) 30.0**

**ALA α-linolenic acid C18:3ꙍ3 (mg/100g) 178.3 Sodium (mg/100g) 46.0**

**Long-chain omega-3s: Potassium (mg/100g) 323.9**

**EPA - C20:5ꙍ3 (mg/100g) 24.2 Copper (mg/100g) 0.1**

**DHA - C22:6ꙍ3 (mg/100g) 8.9 Selenium (mg/100g) 16.0**

**DPA - C25:5ꙍ3 (mg/100g) 25.2**

**EPA + DHA (mg/100g) 33.1**

**EPA + DHA + DPA (mg/100g) 58.3**

**TAW MARGRA lamb cut D**

| **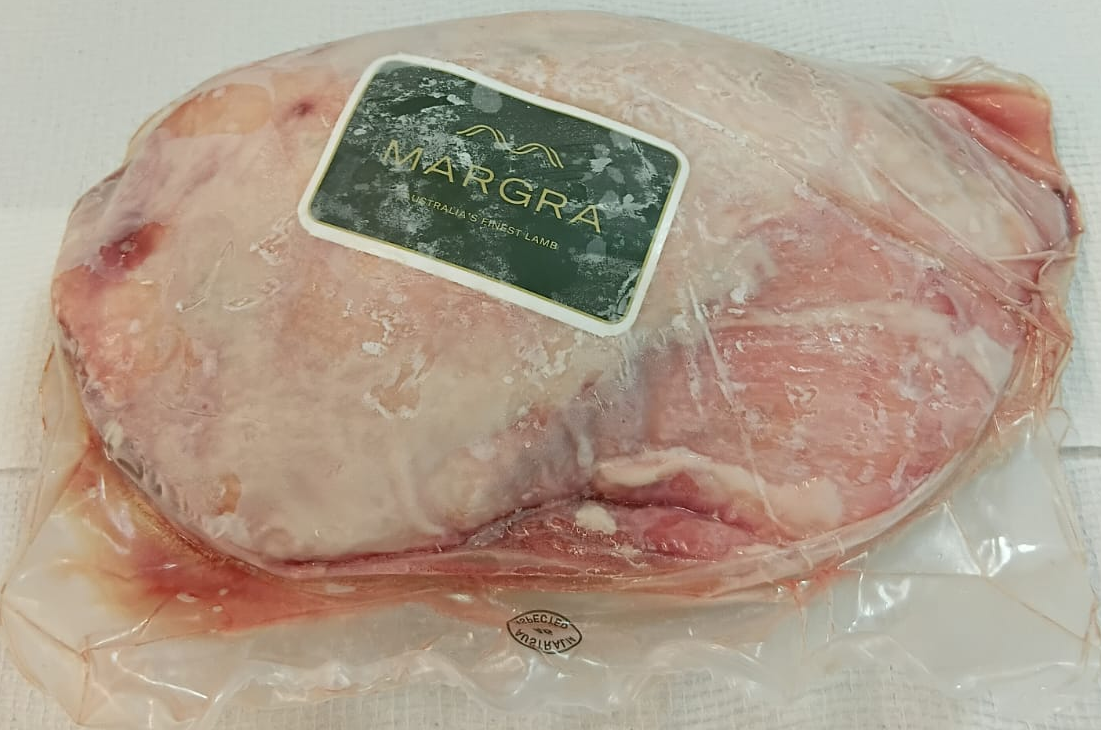** |
| --- |
|  |

**Fat melting point (°C) 35.0 Minerals:**

**Intramuscular fat content (%) 3.3 Iron (mg/100g) 2.8**

**Protein percentage (g/100g) 22.8 Zinc (mg/100g) 2.7**

**Total Fat percentage (g/100g) 14.0 Calcium (mg/100g) 38.9**

**Energy (kJ/100g) 760.9 Phosphorus (mg/100g) 262.0**

**Oleic acid - C18:1ꙍ9 (mg/100g) 2080.1 Magnesium (mg/100g) 31.0**

**ALA α-linolenic acid C18:3ꙍ3 (mg/100g) 99.2 Sodium (mg/100g) 46.2**

**Long-chain omega-3s: Potassium (mg/100g) 326.5**

**EPA - C20:5ꙍ3 (mg/100g) 24.7 Copper (mg/100g) 0.1**

**DHA - C22:6ꙍ3 (mg/100g) 9.3 Selenium (mg/100g) 16.0**

**DPA - C25:5ꙍ3 (mg/100g) 24.4**

**EPA + DHA (mg/100g) 34.0**

**EPA + DHA + DPA (mg/100g) 58.4**

**TAW MARGRA lamb cut E**

| 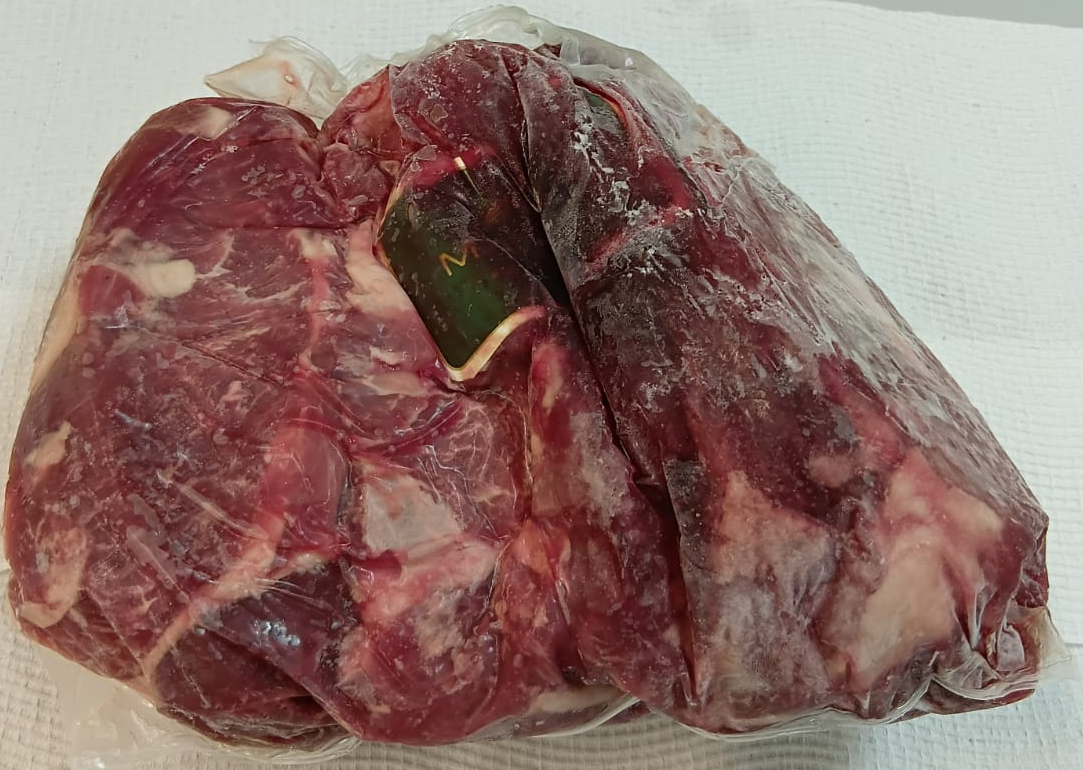 |
| --- |
|  |

**Fat melting point (°C) 38.0 Minerals:**

**Intramuscular fat content (%) 5.5 Iron (mg/100g) 2.7**

**Protein percentage (g/100g) 23.3 Zinc (mg/100g) 2.6**

**Total Fat percentage (g/100g) 6.4 Calcium (mg/100g) 36.9**

**Energy (kJ/100g) 749.2 Phosphorus (mg/100g) 250.3**

**Oleic acid - C18:1ꙍ9 (mg/100g) 3269.4 Magnesium (mg/100g) 31.4**

**ALA α-linolenic acid C18:3ꙍ3 (mg/100g) 179.2 Sodium (mg/100g) 45.8**

**Long-chain omega-3s: Potassium (mg/100g) 323.7**

**EPA - C20:5ꙍ3 (mg/100g) 25.7 Copper (mg/100g) 0.1**

**DHA - C22:6ꙍ3 (mg/100g) 8.5 Selenium (mg/100g) 16.9**

**DPA - C25:5ꙍ3 (mg/100g) 25.7**

**EPA + DHA (mg/100g) 34.2**

**EPA + DHA + DPA (mg/100g) 59.9**

**TAW MARGRA lamb cut F**

| 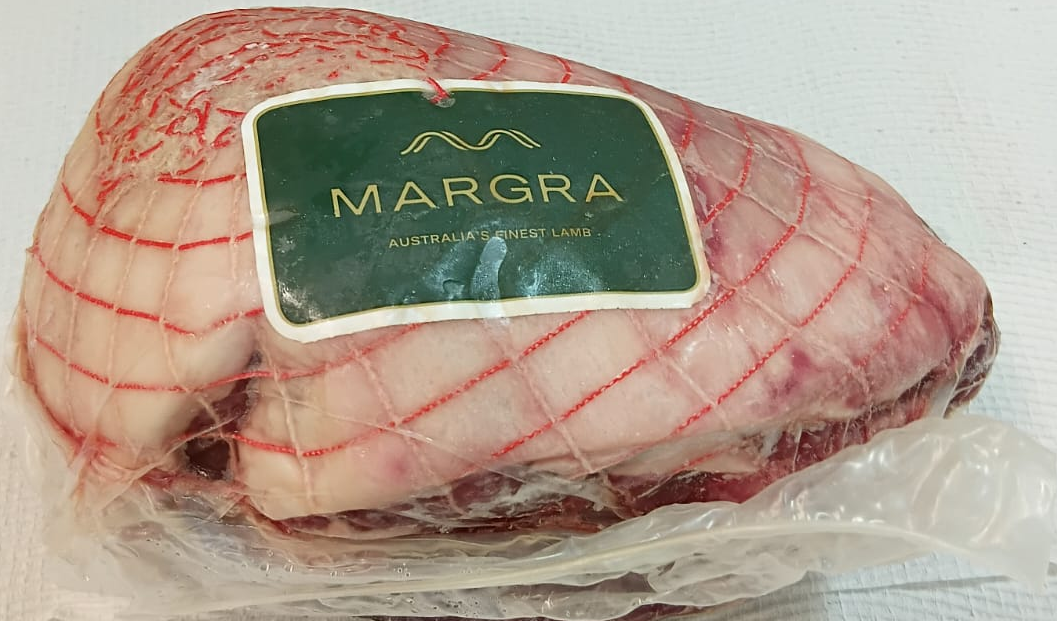 |
| --- |
| **Fat melting point (°C) 37.0 Minerals:**  **Intramuscular fat content (%) 3.5 Iron (mg/100g) 2.6**  **Protein percentage (g/100g) 23.0 Zinc (mg/100g) 2.9**  **Total Fat percentage (g/100g) 14.2 Calcium (mg/100g) 38.0**  **Energy (kJ/100g) 766.4 Phosphorus (mg/100g) 260.3**  **Oleic acid - C18:1ꙍ9 (mg/100g) 2082.7 Magnesium (mg/100g) 30.5**  **ALA α-linolenic acid C18:3ꙍ3 (mg/100g) 99.8 Sodium (mg/100g) 46.4**  **Long-chain omega-3s: Potassium (mg/100g) 322.8**  **EPA - C20:5ꙍ3 (mg/100g) 24.9 Copper (mg/100g) 0.1**  **DHA - C22:6ꙍ3 (mg/100g) 10.0 Selenium (mg/100g) 16.7**  **DPA - C25:5ꙍ3 (mg/100g) 24.1**  **EPA + DHA (mg/100g) 34.9**  **EPA + DHA + DPA (mg/100g) 59.0** |

**TAW MARGRA lamb cut G**

| 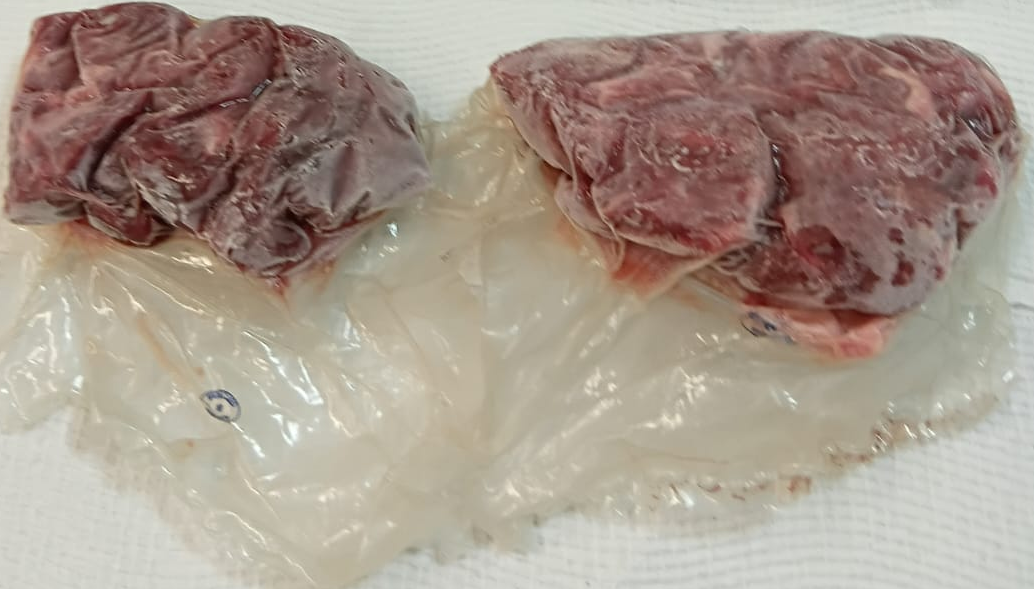 |
| --- |
| **Fat melting point (°C) 36.0 Minerals:**  **Intramuscular fat content (%) 5.0 Iron (mg/100g) 2.8**  **Protein percentage (g/100g) 23.9 Zinc (mg/100g) 2.7**  **Total Fat percentage (g/100g) 6.3 Calcium (mg/100g) 37.9**  **Energy (kJ/100g) 750.5 Phosphorus (mg/100g) 250.8**  **Oleic acid - C18:1ꙍ9 (mg/100g) 3357.4 Magnesium (mg/100g) 31.2**  **ALA α-linolenic acid C18:3ꙍ3 (mg/100g) 180.1 Sodium (mg/100g) 46.8**  **Long-chain omega-3s: Potassium (mg/100g) 322.5**  **EPA - C20:5ꙍ3 (mg/100g) 25.2 Copper (mg/100g) 0.1**  **DHA - C22:6ꙍ3 (mg/100g) 9.5 Selenium (mg/100g) 17.1**  **DPA - C25:5ꙍ3 (mg/100g) 25.3**  **EPA + DHA (mg/100g) 34.7**  **EPA + DHA + DPA (mg/100g) 60.0** |
|  |

**TAW MARGRA lamb cut H**

| 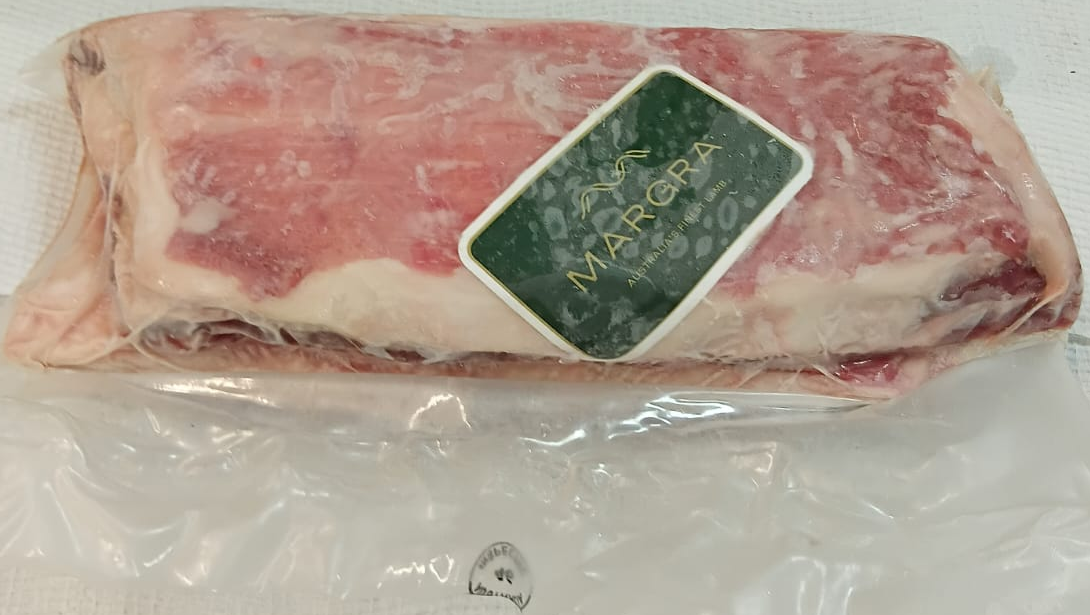 |
| --- |
|  |

**Fat melting point (°C) 37.1 Minerals:**

**Intramuscular fat content (%) 6.3 Iron (mg/100g) 2.7**

**Protein percentage (g/100g) 23.6 Zinc (mg/100g) 2.8**

**Total Fat percentage (g/100g) 14.3 Calcium (mg/100g) 38.4**

**Energy (kJ/100g) 760.5 Phosphorus (mg/100g) 259.9**

**Oleic acid - C18:1ꙍ9 (mg/100g) 2088.2 Magnesium (mg/100g) 31.5**

**ALA α-linolenic acid C18:3ꙍ3 (mg/100g) 98.6 Sodium (mg/100g) 45.9**

**Long-chain omega-3s: Potassium (mg/100g) 322.9**

**EPA - C20:5ꙍ3 (mg/100g) 25.2 Copper (mg/100g) 0.1**

**DHA - C22:6ꙍ3 (mg/100g) 9.9 Selenium (mg/100g) 16.6**

**DPA - C25:5ꙍ3 (mg/100g) 24.3**

**EPA + DHA (mg/100g) 36.1**

**EPA + DHA + DPA (mg/100g) 60.4**

**MINCED TAW MARGRA (PATTIES)**

**Fat melting point (°C) 36.6 Minerals:**

**Intramuscular fat content (%) 6.3 Iron (mg/100g) 2.7**

**Protein percentage (g/100g) 23.8 Zinc (mg/100g) 2.8**

**Total Fat percentage (g/100g) 6.4 Calcium (mg/100g) 38.0**

**Energy (kJ/100g) 750.8 Phosphorus (mg/100g) 250.6**

**Oleic acid - C18:1ꙍ9 (mg/100g) 3358.0 Magnesium (mg/100g) 31.0**

**ALA α-linolenic acid C18:3ꙍ3 (mg/100g) 180.0 Sodium (mg/100g) 47.9**

**Long-chain omega-3s: Potassium (mg/100g) 322.7**

**EPA - C20:5ꙍ3 (mg/100g) 25.0 Copper (mg/100g) 0.1**

**DHA - C22:6ꙍ3 (mg/100g) 9.4 Selenium (mg/100g) 17.0**

**DPA - C25:5ꙍ3 (mg/100g) 24.9**

**EPA + DHA (mg/100g) 34.4**

**EPA + DHA + DPA (mg/100g) 59.3**
