# Supplementary material for: Validation of stearoyl-coA desaturase gene TaqMan probe-based SNP for genotyping Tattykeel Australian White MARGRA lamb for health-beneficial omega-3 long-chain fatty acids and intramuscular fat content
Source: PLoS One. 2026 Jan 6;21(1):e0339573. doi: 10.1371/journal.pone.0339573 (PMC12773820; doi:10.1371/journal.pone.0339573)
Supplement: Attachment — Submitted filename: Reviewer Report PONE-D-25-49554.pdf [file pone.0339573.s003.pdf]

Reviewer Report: PONE-D-25-49554
Manuscript Title: [**Validation of a TaqMan probe-based SNP genotyping for the sheep stearoyl-coA desaturase gene in Tattykeel Australian White MARGRA Lamb: Implications for health beneficial omega-3 long-chain polyunsaturated fatty acids and intramuscular fat content**]
Journal: [**PLOS ONE**]

Reviewer Report

Summary of the Research and Overall Impression

This manuscript describes the development and validation of a TaqMan probe-based Real-Time PCR assay for the genotyping of a single nucleotide polymorphism (SNP; g.23881050T>C) in the Stearoyl-CoA Desaturase (SCD) gene of Tattykeel Australian White (TAW) sheep. The SCD gene is directly involved in fatty acid metabolism and intramuscular fat deposition, traits that are strongly associated with meat quality and consumer preference.

The study is timely and relevant, as it addresses a key limitation in livestock breeding by providing a cost-effective, rapid, and accurate genotyping tool. The authors further demonstrate the assay's applicability across breeds and include cattle as an interspecies control, which adds robustness to their validation. The findings have practical implications for integrating molecular markers into selective breeding programs aimed at improving eating quality in TAW sheep.

Strengths of the study include its clear objectives, practical focus on an economically important trait, and a well-documented methodology. However, the manuscript also presents some limitations. The population size is relatively small, potentially limiting the generalizability of the allele frequency estimates. Moreover, the absence of the TT genotype requires further discussion, and additional clarification of methodological and statistical details would strengthen the work.

Overall, the manuscript is of good quality and presents valuable results for animal genetics and breeding. I recommend minor to moderate revisions before acceptance.

2. Specific Areas for Improvement
Major Issues

Absence of TT Genotype (Lines 360–362)
The manuscript reports only CC and CT genotypes in the TAW population, with no observation of TT. This should be discussed in greater depth. Possible explanations include selective breeding practices, low minor allele frequency, or genetic drift. A brief quantitative evaluation or reference to prior reports would strengthen this point.

Sample Size and Representativeness (Lines 153–161)
The study involves 113 animals plus controls, which may not fully capture the genetic diversity of the TAW population. The authors should acknowledge this limitation and indicate that further validation in larger and more diverse cohorts is warranted.

Functional Relevance of the SNP (Lines 127–133, 293–301)
While the study highlights an association between the SNP and meat quality traits, the functional mechanisms underlying this relationship are not elaborated. Additional discussion of SCD gene variants and their role in fatty acid metabolism in ruminants would add depth and biological relevance.

Limitations of the TaqMan Assay (Lines 111–125)
Although TaqMan technology is robust, the manuscript should address potential limitations, such as allele dropout, probe specificity, or difficulties in detecting rare alleles. Acknowledging these will provide a more balanced view of the method.

Interpretation of Allele Frequencies (Lines 260–277)
The differences in allele frequencies among breeds are well presented but would benefit from more critical discussion. How might these patterns influence long-term breeding strategies, genetic diversity, and the risk of inbreeding depression?

Minor Issues

Figure and Table Integration
Figures 1 and 2 clearly illustrate the genotyping results. However, the manuscript should more explicitly reference these figures in the results section to guide readers through the key findings.

Reference Consistency
Some references are repeated with slight variations in formatting (e.g., Lines 4, 11, 39). A thorough check for consistency with journal guidelines is recommended.

Terminology and Style

Certain phrases (e.g., “high-specificity,” “rapid genotyping”) are used repeatedly. Standardizing terminology will improve readability. Additionally, some sentences in the introduction are overly long and could be streamlined for clarity.

Abbreviations

Abbreviations such as IMF, FMP, and LC-PUFA should be defined upon first use and applied consistently throughout the text.

3. Conclusion

This study provides a valuable contribution to the field of livestock genomics and demonstrates the potential of molecular tools in selective breeding programs for meat quality improvement. The methodology is sound, the findings are relevant, and the practical application is clear.

Addressing the major issues—particularly regarding genotype absence, sample size, functional interpretation, and assay limitations—will enhance the manuscript’s scientific rigor and impact. Minor adjustments in style, references, and figure integration will improve clarity and presentation.
