# Supplementary material for: Validation of stearoyl-coA desaturase gene TaqMan probe-based SNP for genotyping Tattykeel Australian White MARGRA lamb for health-beneficial omega-3 long-chain fatty acids and intramuscular fat content
Source: PLoS One. 2026 Jan 6;21(1):e0339573. doi: 10.1371/journal.pone.0339573 (PMC12773820; doi:10.1371/journal.pone.0339573)
Supplement: Attachment — Submitted filename: Response2 to Reviewers Report PONE-D-25-49554.docx [file pone.0339573.s006.docx]

**RESPONSE2 TO REVIEWER REPORT: PONE-D-25-49554**

**Manuscript Title:** [**Validation of a TaqMan probe-based SNP genotyping for the sheep stearoyl-coA desaturase gene in Tattykeel Australian White MARGRA Lamb: Implications for health beneficial omega-3 long-chain polyunsaturated fatty acids and intramuscular fat content**]

**Journal:** [**PLOS ONE**]

**-----------------------------------------------------------------------------------------------------------**

**REVIEWERS’** **RESPONSES TO QUESTIONS**

**Comments to the Author**

1. If the authors have adequately addressed your comments raised in a previous round of review and you feel that this manuscript is now acceptable for publication, you may indicate that here to bypass the “Comments to the Author” section, enter your conflict of interest statement in the “Confidential to Editor” section, and submit your "Accept" recommendation.

Reviewer #1: All comments have been addressed

Reviewer #2: All comments have been addressed

2. Is the manuscript technically sound, and do the data support the conclusions?

Reviewer #1: Yes

Reviewer #2: Yes

3. Has the statistical analysis been performed appropriately and rigorously?

Reviewer #1: Yes

Reviewer #2: Yes

4. Have the authors made all data underlying the findings in their manuscript fully available?

Reviewer #1: Yes

Reviewer #2: Yes

5. Is the manuscript presented in an intelligible fashion and written in standard English?

Reviewer #1: Yes

Reviewer #2: Yes

**6. Review Comments to the Author**

**Reviewer #1**: After reviewing the revised manuscript and the authors’ detailed responses, I confirm that all major and minor comments have been thoroughly addressed. The authors substantially improved the discussion of the missing TT genotype, supported by appropriate references and a clearer explanation of selective breeding and allele frequency dynamics. The inclusion of a dedicated Limitations section strengthens the transparency of the study, particularly regarding sample size and the constraints of the TaqMan assay.

The expanded discussion on the functional relevance of the SCD gene and its role in fatty acid metabolism enhances the biological interpretation of the findings. Minor issues related to terminology, reference formatting, figure integration, and abbreviation consistency have been adequately corrected.

Overall, the manuscript has improved significantly in clarity, scientific rigor, and presentation. I recommend acceptance after minor editorial proofreading.

Reviewer #2: Most of the previous comments are correctly addressed in the manuscript, although introductory lines of the abstract still seems lengthy.

**RESPONSE: We are grateful to both reviewers for their second round comments. We have made some minor editorial proofreading changes and cut back on the lengthy introductory lines.**
